# Childhood trauma and the architecture of attachment in first-episode psychosis

**DOI:** 10.1192/j.eurpsy.2026.12169

**Published:** 2026-07-31

**Authors:** S. Hamzaoui, K. Mahfoudh, S. Walha, R. Lansari, A. Larnaout

**Affiliations:** Department of psychiatry D, Razi Hospital, Manouba, Tunisia

## Abstract

**Introduction:**

Childhood trauma is a major disruptor of attachment. In psychosis, insecure attachment worsens outcomes, but its link with trauma in first-episode psychosis (FEP) is less documented.

**Objectives:**

To explore associations between childhood maltreatment and attachment styles in stabilized FEP patients.

**Methods:**

We studied 60 outpatients with FEP stabilized for at least 2 months. Childhood trauma was assessed with the Childhood Trauma Questionnaire–Short Form (CTQ-SF), which evaluates five domains of maltreatment: emotional abuse, physical abuse, sexual abuse, emotional neglect, and physical neglect. Attachment was measured with the Experiences in Close Relationships–Revised (ECR-R), assessing two main dimensions—attachment anxiety and attachment avoidance—allowing classification into four attachment styles: secure, anxious, fearful, and dismissing. Symptom severity was evaluated with the Positive and Negative Syndrome Scale (PANSS).

**Results:**

The effects of childhood trauma on attachment showed significant results. The total CTQ score and its subscales—emotional abuse, physical abuse, sexual abuse, and physical neglect—were significantly and positively correlated with insecure attachment. The total CTQ score was significantly and positively correlated with attachment anxiety and attachment avoidance. Emotional abuse was correlated with attachment anxiety and fearful attachment. Physical abuse, physical neglect, and emotional neglect were correlated with attachment avoidance. Sexual abuse was significantly and positively correlated with dismissing attachment.

**Conclusions:**

Childhood trauma is a robust predictor of insecure attachment in FEP, with differential effects depending on maltreatment type. These findings highlight the need for trauma-informed assessment and attachment-focused interventions in early psychosis care.

**Disclosure of Interest:**

None Declared

